# Correlative High-Resolution Imaging of Iron Uptake in Lung Macrophages

**DOI:** 10.1021/acs.analchem.2c02675

**Published:** 2022-09-07

**Authors:** Jelena Lovrić, Neda Najafinobar, Michael E. Kurczy, Olivier De Castro, Antje Biesemeier, Lena von Sydow, Magnus Klarqvist, Tom Wirtz, Per Malmberg

**Affiliations:** † DMPK, Research and Early Development, Cardiovascular, Renal and Metabolism, BioPharmaceuticals R&D, AstraZeneca, SE-431 50 Gothenburg, Sweden; ‡ Medicinal Chemistry, Research and Early Development, Respiratory and Immunology, BioPharmaceuticals R&D, AstraZeneca, SE-431 50 Gothenburg, Sweden; § Advanced Instrumentation for Nano-Analytics (AINA), MRT Department, Luxembourg Institute of Science and Technology (LIST), L-4422 Belvaux, Luxembourg; ∥ Early Product Development, Pharm Sci, IMED Biotech Unit, AstraZeneca, SE-431 50 Gothenburg, Sweden; ⊥ Department of Chemistry and Chemical Engineering, Chalmers University of Technology, SE-412 96 Gothenburg, Sweden

## Abstract

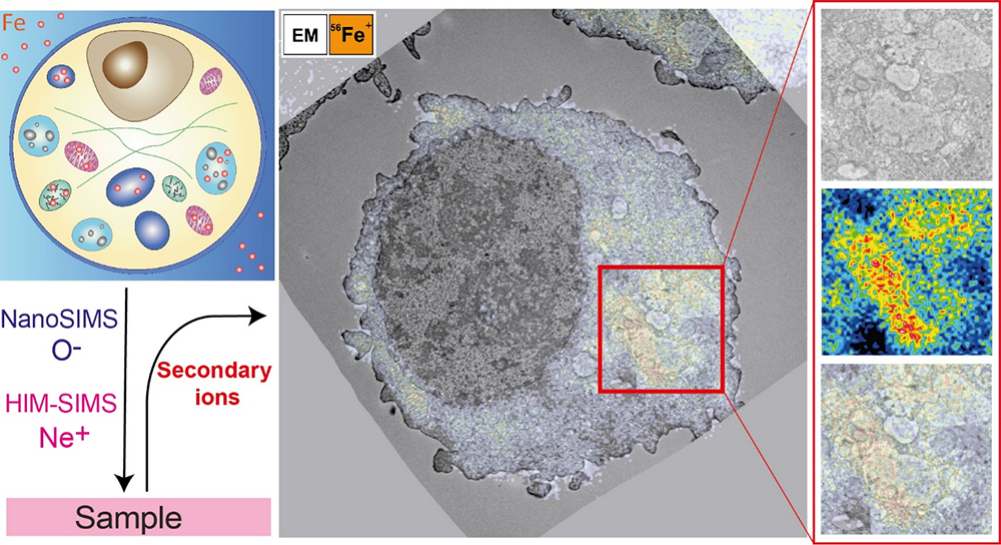

Detection of iron at the subcellular level in order to gain insights into its transport, storage, and therapeutic prospects to prevent cytotoxic effects of excessive iron accumulation is still a challenge. Nanoscale magnetic sector secondary ion mass spectrometry (SIMS) is an excellent candidate for subcellular mapping of elements in cells since it provides high secondary ion collection efficiency and transmission, coupled with high-lateral-resolution capabilities enabled by nanoscale primary ion beams. In this study, we developed correlative methodologies that implement SIMS high-resolution imaging technologies to study accumulation and determine subcellular localization of iron in alveolar macrophages. We employed transmission electron microscopy (TEM) and backscattered electron (BSE) microscopy to obtain structural information and high-resolution analytical tools, NanoSIMS and helium ion microscopy-SIMS (HIM-SIMS) to trace the chemical signature of iron. Chemical information from NanoSIMS was correlated with TEM data, while high-spatial-resolution ion maps from HIM-SIMS analysis were correlated with BSE structural information of the cell. NanoSIMS revealed that iron is accumulating within mitochondria, and both NanoSIMS and HIM-SIMS showed accumulation of iron in electrolucent compartments such as vacuoles, lysosomes, and lipid droplets. This study provides insights into iron metabolism at the subcellular level and has future potential in finding therapeutics to reduce the cytotoxic effects of excessive iron loading.

## Introduction

Iron is an essential trace element that plays an important role in oxygen transport and energy production in the cell.^1^ Within the cell, iron can be utilized for synthesis of heme and iron–sulfur clusters^2^ or be directly bound to proteins such as ferritin, hemosiderin, transferrin,^3^ and lactoferrin^4^ that are localized in subcellular compartments such as cytoplasm, lysosomes,^5^ mitochondria,^2^ or vacuoles.^6^ In fact, due to its sheer complexity, intracellular iron metabolism has been compared to a symphony orchestra.^7^ It is well known that disruptions within the iron metabolism can be detrimental, e.g., excessive free iron in cells is known to generate free radicals, which may eventually cause cellular and genetic damage.^8^ Excessive iron accumulation has been observed in several different diseases such as neurodegenerative diseases^9^ and chronic hepatitis C.^10^ Diseases in the lung are especially of interest since the airways are directly affected by atmospheric iron sources such as cigarette smoke and airborne particulate matter.^11^ Increased intracellular iron levels have also been demonstrated in chronic obstructive pulmonary disease (COPD).^12^ COPD alveolar macrophages exhibit increased iron levels, and an increased percentage of iron-positive macrophages with increased COPD severity has also been demonstrated.^13^ In cells, mitochondria are the main consumers of iron. Mitochondrial dysregulation of iron handling has been observed in cigarette smoke-induced bronchitis and emphysema in mice.^14^ Despite many studies regarding iron metabolism in disease at the subcellular level, much remains to be done when it comes to determining the exact subcellular localization. This can contribute to understanding potential therapeutic opportunities to reduce the effects of excessive iron loading.

Tracing of intracellular iron is predominately done by histochemical methods such as Perls’ Prussian blue staining, a stain that is usually employed to visualize and quantify iron in cells and lung tissue sections.^14^ Iron staining is difficult since environmental particulates such as anthracotic material also stain with Perls’ staining.^15^ X-ray fluorescence microscopy is a viable alternative for mapping intracellular iron,^16^ yet obtaining true subcellular localization has to be demonstrated for iron in cells.

Secondary ion mass spectrometry (SIMS) imaging has been successfully utilized to analyze iron in biological samples.^17−19^ It provides an acquisition of secondary ion maps showing the elemental or molecular distribution in a sample. In SIMS analysis, the surface of a sample is bombarded with an accelerated focused primary ion beam. During this process, material is sputtered away and secondary ion species are generated, analyzed in a mass analyzer, and detected as a mass spectrum for each pixel in an image. With a detection limit in the parts per million or even parts per billion range and its high mass- and spatial-resolution capabilities, the SIMS technique is highly applicable for analysis of different materials such as semiconductors,^20^ geological and cosmological samples,^21^ metals,^22^ and biological samples.^23−25^ In a previous study, we showed the potential of time-of-flight SIMS (ToF-SIMS) to visualize and relatively quantify iron accumulation in human lung tissue sections.^26^ This analysis revealed a heterogeneous distribution of iron within cells, consistent with iron accumulation in discrete cellular organelles such as mitochondria,^2^ lysosomes,^5^ or vacuoles.^6^ Although imaging with ToF-SIMS is highly beneficial as it allows to follow all secondary ions in parallel with molecular information included, many ToF-SIMS instruments show dependency between spatial-resolution capability and mass-resolution capability, as the primary ion beam pulses have to be compressed to increase mass resolving power. This leads to deterioration of the primary ion beam spot size and consequently lateral resolution of the instrument.^27,28^ Therefore, in the abovementioned study, the exact subcellular information, a much needed piece of information in understanding iron metabolism, was not provided. A double-focusing magnetic sector mass analyzer (a combination of electrostatic and magnetic sectors to reach achromatic mass filtering)^29^ can also be used to study a subcellular distribution of analytes. The Cameca NanoSIMS is a high-spatial, high-mass-resolution double-focusing SIMS instrument often applied in biological studies. The instrument employs reactive oxygen or cesium primary ion species to sputter away positive or negative secondary ions, respectively. The NanoSIMS instrument has coaxial primary and secondary ion optics^30^ allowing spatial-resolution imaging down to 50 nm and can detect seven secondary ion species in parallel. However, due to a high fragmentation rate at high irradiation doses, molecular information is lost and only monoatomic or small cluster secondary ions are generated. Therefore, it is necessary to use elemental or isotopic labels to detect molecules of interest. Besides NanoSIMS, a double-focusing magnetic sector mass analyzer, helium ion microscope secondary ion mass spectrometer (HIM-SIMS),^31^ was developed for high-spatial-resolution chemical imaging. It is an in situ ion microscope and mass spectrometer that employs a gas field ion source.^32,33^ This source generates noble gas ions such as He^+^ or Ne^+^ as a primary ion species. While the He^+^ beam is mostly used for secondary electron imaging, the Ne^+^ beam is most appropriate for patterning and ion imaging in SIMS operation mode. A big advantage of the HIM-SIMS instrument is the ultimate spatial resolution in the SIMS mode, allowing analyses down to the sub-20 nm. The probe size for the Ne ion beam is 2 nm, which is considerably smaller than the secondary ion emission area; hence, the lateral resolution is not limited anymore by the probe size but only by the size of the collision cascade in a sample.^34,35^ It is possible to image four ion species in parallel with the older version of the HIM-SIMS detection system that was used in this study (a multicollector with one fixed and three movable electron multipliers).^36^ A newer detection system relying on a continuous focal plane detector (microchannel plate array) allows acquisition of a full mass spectrum for each pixel.^37^ The HIM-SIMS is still considered somewhat of a new technology in the area of life sciences,^38^ and only a few studies employed HIM-SIMS imaging methodology to study for example distribution of nanoparticles and nanowires in biological systems^39−41^ or to localize perfluorooctanoic acid in tissue and cells at the subcellular level.^42^

The goal of this study was to develop methodologies to analyze elemental iron in alveolar macrophages at the subcellular level using nanoscale SIMS. First, the structural information of a sample was obtained with a transmission electron microscope (TEM) or scanning electron microscope (SEM) in order to obtain regions of interest (ROIs) for subsequential chemical imaging. We then demonstrated high-spatial-resolution imaging of iron uptake in alveolar macrophages by using NanoSIMS and HIM-SIMS techniques that both provided subcellular localization of targeted elements. The chemical signal of iron was correlated with structural information obtained from TEM in the case of NanoSIMS and SEM in the case of HIM-SIMS analysis. We could show a clear localization of iron in subcellular structures such as mitochondria and vacuole-like organelles demonstrating the usefulness of the techniques for subcellular iron imaging.

## Experimental Section

### Materials

#### Cell Culture and Treatment

NR8383 cells, an immortalized cell line derived from lung macrophages (*Rattus* sp.), was purchased from ATCC (USA). Uncoated 35 mm dishes with a glass coverslip were obtained from MatTek Life Sciences (USA). DMEM/F-12 GlutaMAX supplement, Opti-MEM Reduced Serum Medium, and 15% dialyzed heat-inactivated fetal bovine serum were purchased from Gibco (USA). Ammonium iron(III) citrate and bovine serum albumin (BSA) were obtained from Sigma-Aldrich (USA). Water was purified with a Milli-Q IQ 7003 purification system (Merck, Germany).

#### Chemical Fixation

Glutaraldehyde, sodium cacodylate, and Agar 100 resin were purchased from Agar Scientific (UK). Sodium azide was obtained from BDH (UK). Osmium tetroxide and formaldehyde were purchased from Sigma-Aldrich (USA). Uranyl acetate was obtained from Merck (Germany). Formvar-coated copper grids were purchased from EMS (USA).

### Methods

#### Cell Culturing and Drug Incubation

NR8383 cells were cultured in uncoated dishes in DMEM/F-12, GlutaMAX supplement, and 15% dialyzed, heat-inactivated fetal bovine serum. Cells were seeded at a cell density of 2 × 10^5^ cells/mL and cultured for 3 days prior to exposure with ammonium iron(III) citrate. A 5 mM stock solution of ammonium iron(III) citrate was prepared in ultrapure water. A working solution was prepared by diluting a stock solution in Opti-MEM Reduced Serum Medium supplemented with 0.1% BSA to obtain 500 μM solution of ammonium iron(III) citrate. Macrophages were treated for 72 h in an incubator at 37 °C in a water-saturated atmosphere containing 5% CO_2_.

#### Chemical Fixation

The NR8383 cells were incubated with a modified Karnovsky fixative^43^ containing 2% formaldehyde, 2.5% glutaraldehyde, 50 mM sodium cacodylate buffer, and 0.01% sodium azide for 30 min at room temperature. Cells were washed with 100 mM sodium cacodylate buffer and postfixed with 1% osmium tetroxide at room temperature for 2 h in the dark. This was followed by exposure with 1% tannic acid for 20 min in the dark. Afterward, cells were treated with 1% uranyl acetate for 15 min at room temperature in the dark. Dehydration was performed using rising concentrations of ethanol (50, 70, 85, 95, and 99.5%). Embedding was done in Agar 100 resin following manufacturer formulation to obtain a medium-hard resin block. Sections were cut using a Leica EM UC6 ultramicrotome. TEM imaging was done on 70 nm thick sections placed on formvar-coated copper grids. Sequential sections of 300 nm thickness were placed on the same type of TEM grid for NanoSIMS analysis (Figure S1). Post-staining of the samples using uranyl acetate and Reynolds lead citrate^44^ was performed directly on the grids. Backscattered electron (BSE) imaging followed by HIM-SIMS analysis was done on 500 nm thick sections placed on Si wafers without post-staining.

#### Transmission Electron Microscopy

Electron microscopy observations were carried out with Leo 912AB Ω (2k × 2k Veleta CCD camera, Carl Zeiss) and TALOS L120C (4k × 4k Ceta-M CMOS camera, Thermo Fischer Scientific) TEMs operated at 120 kV.

#### BSE Microscopy

The BSE images were acquired with a focused ion beam scanning electron microscope (Scios DualBeam, Thermo Fisher Scientific, Eindhoven, Netherlands) and a field emission scanning electron microscope (ZEISS Gemini 500, Carl Zeiss, Oberkochen, Germany) operating under high-vacuum conditions. BSE imaging was carried out at a primary electron energy of 5 keV in a matrix of 3072 × 2048 pixels and 2048 × 1536 pixels and a horizontal field width (HFW) between 15 and 28 μm. Samples were gold-coated (approx. 5–6 nm thick layer) prior to BSE imaging.

#### NanoSIMS Analysis

**Figure 1:**
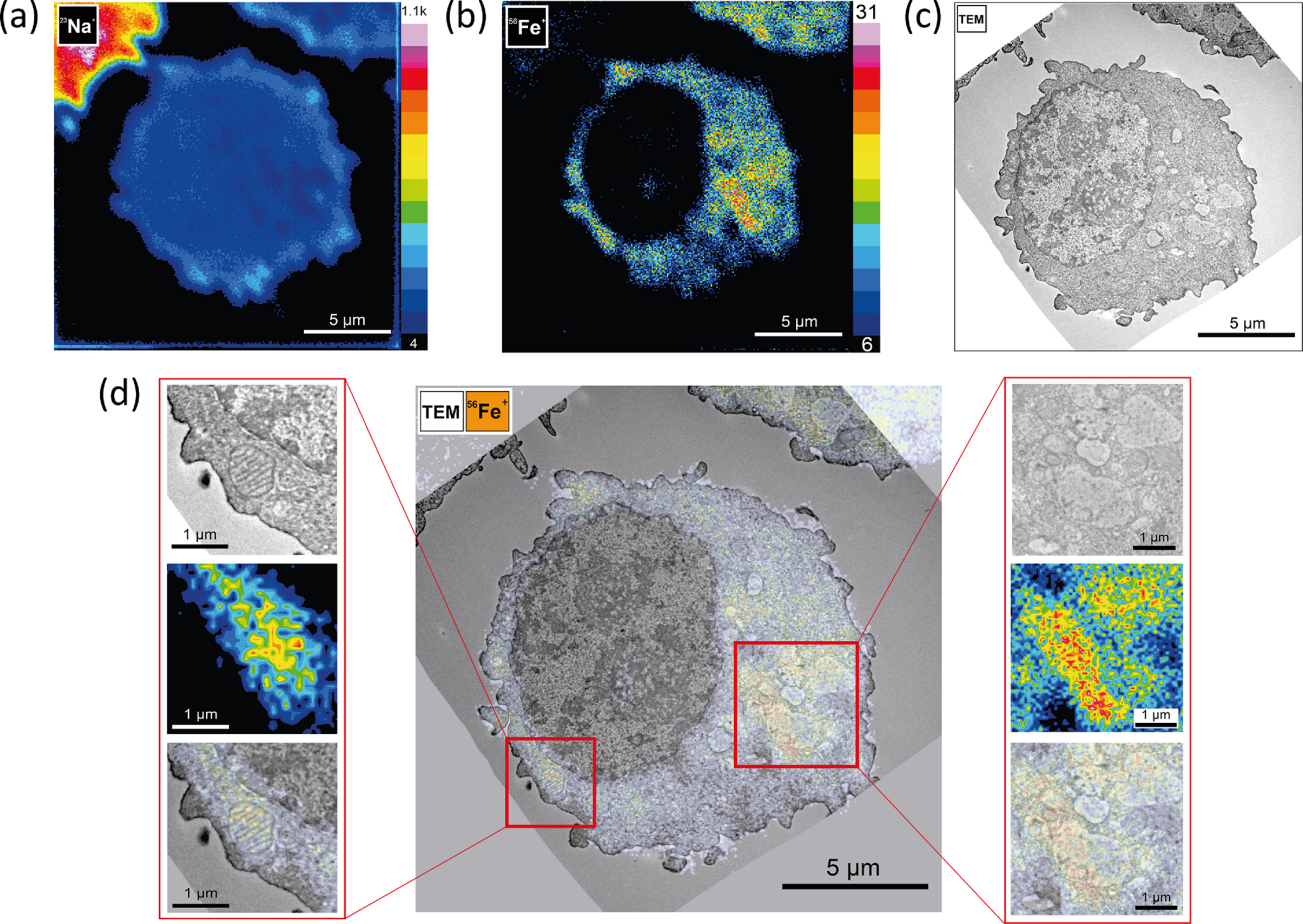
Correlative TEM and NanoSIMS imaging of iron in alveolar macrophages exposed to 500 μM ammonium iron(III) citrate and chemically fixed. NanoSIMS imaging: (a) ion map of ^23^Na^+^ revealing a cellular contour; (b) ^56^Fe^+^ ion map. For both NanoSIMS images: 16 keV O^–^, fluence: 36.11 × 10^16^ ions/cm^2^, FoV: 20 × 20 μm^2^, number of planes: 41; (c) corresponding TEM image; (d) an overlay of TEM image and ^56^Fe^+^ signal to correlate structural and chemical information. A blow-up of two ROIs from TEM image, ^56^Fe^+^ ion map and from their overlay, showing localization of iron in mitochondria (on left) and vacuole-like organelles (on the right). Scale bars: 1 and 5 μm.

**Figure 2:**
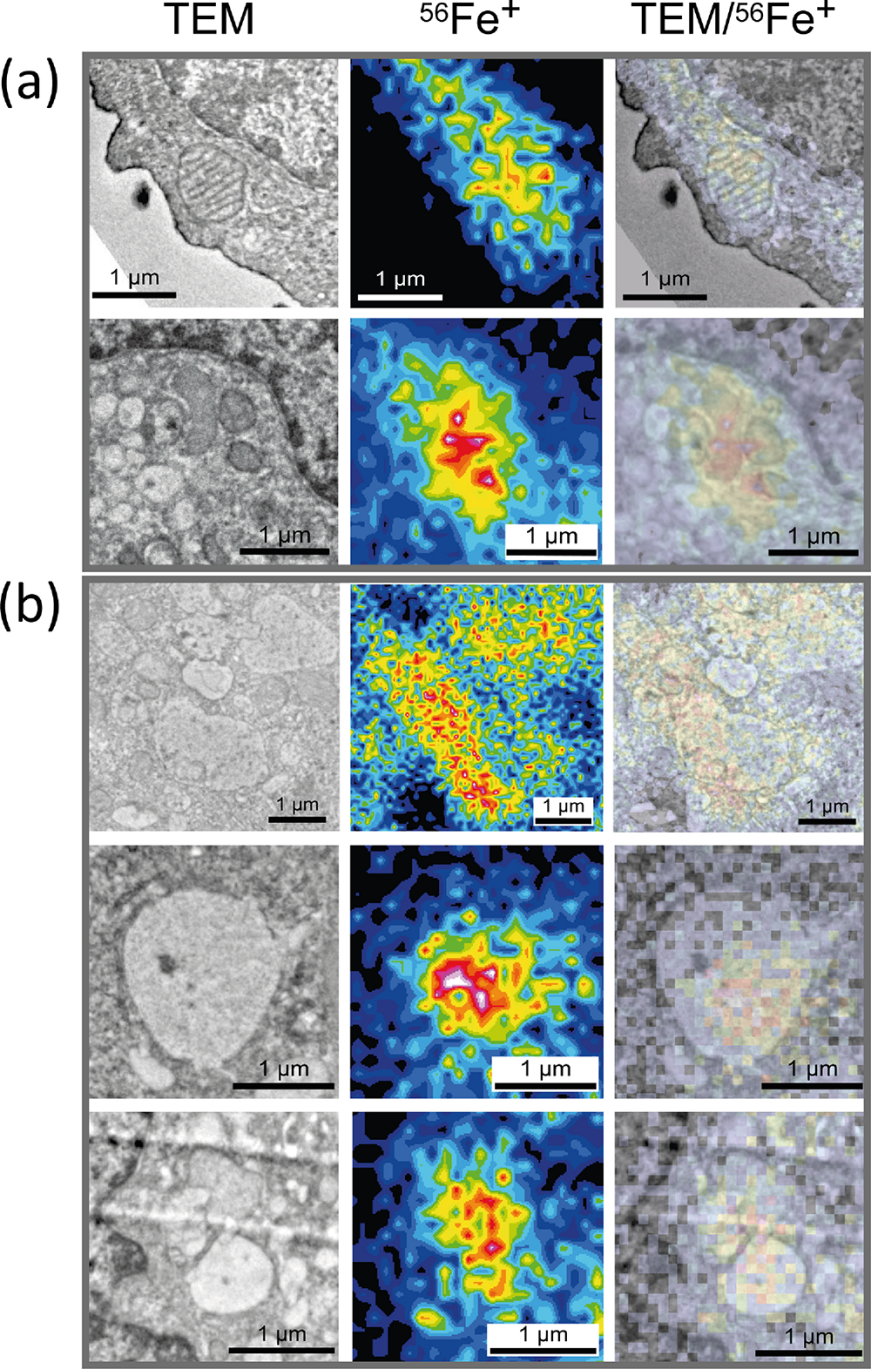
A summary of selected ROIs from three analyzed cells treated with 500 μM ammonium iron(III) citrate and later chemically fixed. From left to right: TEM image, ^56^Fe^+^ ion image, and their overlay. ROIs selected in NanoSIMS images were acquired from ^56^Fe^+^ ion maps with a size of 2048 × 2048 pixels (for more details, see Figures S4 and S5). (a) Sequestration of iron in mitochondria; (b) sequestration of iron in vacuole-like organelles. Scale bar: 1 μm*.*

High-resolution SIMS images were acquired using a NanoSIMS 50L instrument (Cameca, Gennevilliers, France). Analysis was performed using the duoplasmatron oxygen primary ion source generating positive secondary ion species. The impact energy of negatively charged primary ions was 16 keV. Prior to imaging, samples were gold-coated and implantation of oxygen ions was done by scanning the area of interest with a defocused primary ion beam with a high primary ion current (∼130 pA) in order to increase the ionization yield of secondary ions (SI). The instrument was tuned for C^+^ (*m*/*z =* 12), Na^+^ (*m*/*z =* 23), P^+^ (*m*/*z =* 31), and Fe^+^ (*m*/*z =* 56) ions to give morphological information and the distribution of iron. The mass resolution was optimized in order to resolve mass interference occurring for iron (*m*/Δ*m* ∼ 2500 to resolve ^40^Ca^16^O^+^ and ^56^Fe^+^). NanoSIMS images were collected with a dwell time of 5 ms/pixel, at 256 × 256 pixels, with aperture diaphragm slit D1–2 (300 μm) and with raster sizes of 20 × 20 and 30 × 30 μm^2^. Image analysis was done using Fiji open-source software^45^ with OpenMIMS plugin (v3.0.5, 2018 (rev: 1); MIMS, Harvard University; http://www.nrims.hms.harvard.edu/). Images were drift-corrected, and final ion maps typically contained 5–40 image planes. SIMS images of the selected ROIs shown in Figures 1 and 2 and Figures S1 and S2 were obtained from ion images previously scaled to 2048 × 2048 pixels (see Figures S4 and S5 for more details).

#### HIM-SIMS Analysis

HIM imaging in the secondary electron mode was carried out using a He^+^ ion beam at an energy of 20 keV in a matrix of 1024 × 1024 pixels with a scan dwell time of 10 μs and a field of view (FoV) between 14 × 14 and 20 × 20 μm^2^. Samples were gold-coated (approx. 5–6 nm thick layer) prior to HIM and HIM-SIMS imaging. High-resolution secondary ion images were acquired with a SIMS system developed at LIST for the ORION NanoFab Helium Ion Microscope (ZEISS, USA).^46,47^ The spectrometer was tuned for simultaneous detection of Na^+^ (*m*/*z =* 23), Ca^+^ (*m*/*z =* 40), and Fe^+^ (*m*/*z =* 56) signals in the positive polarity. CN^–^ (*m*/*z =* 26) and Cl^–^ (*m*/*z =* 35) secondary ions were detected in the negative polarity. The ion images were acquired with a Ne^+^ primary ion beam at a beam energy of 20 keV in a matrix of 512 × 512 pixels with a dwell time of 3 ms/pixel. The sample voltage was −500 V to extract negative SI and +500 V to obtain positive SI. The SI were then post-accelerated to 3.5 keV in the spectrometer. In the positive polarity, the FoV was in a range between 14 × 14 and 20 × 20 μm^2^ and the ion fluence was between 0.74 × 10^16^ and 2.2 × 10^16^ ions/cm^2^. In the negative polarity, the FoV was in a range between 12 × 12 and 18 × 18 μm^2^ and the ion fluence was between 0.85 × 10^16^ and 1.86 × 10^16^ ions/cm^2^. Image analysis was done using Fiji open-source software with OpenMIMS plugin (v3.0.5, 2018 (rev: 1); MIMS, Harvard University). Post-acquisition image processing was performed on iron and calcium signals as binning (X,Y shrink factor: 2; bin method: sum) in order to enhance contrast. However, this transformation affected the lateral resolution.

#### Alignment of SIMS and EM Images

**Figure 3:**
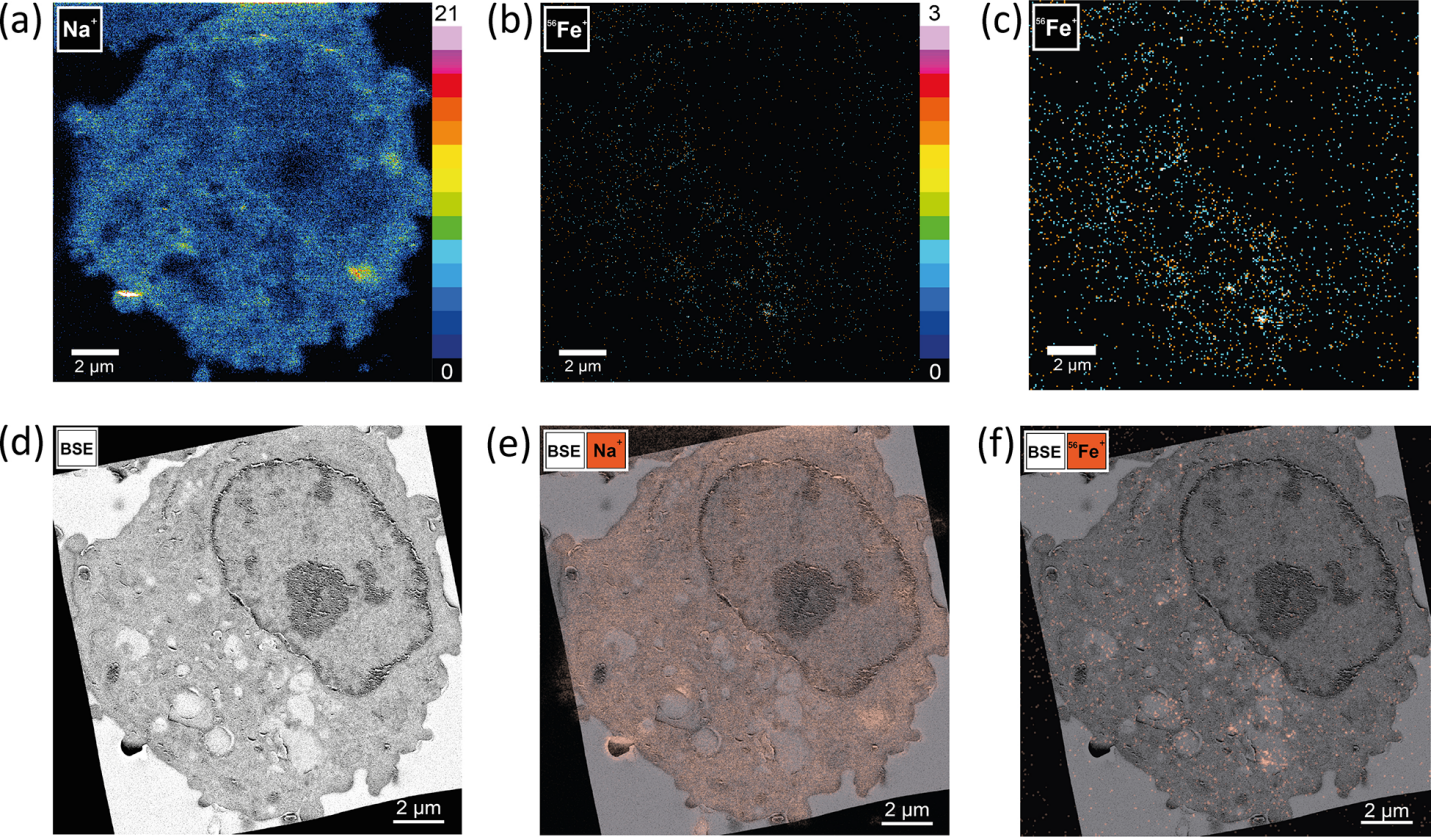
Correlative BSE and HIM-SIMS imaging of iron in chemically fixed alveolar macrophages previously treated with 500 μM ammonium iron(III) citrate. HIM-SIMS ion images: (a) ^23^Na^+^ revealing a cellular contour; (b) ^56^Fe^+^ ion map; (c) binned ^56^Fe^+^ ion signal (X,Y shrink factor: 2; bin method: sum); (d) BSE image of the same cell; (e) overlay of the ^23^Na^+^ SIMS signal and BSE structural information; (f) overlay of the binned ^56^Fe^+^ ion signal and BSE structural information revealing localization of iron in vacuole-like organelles. HIM-SIMS imaging: 20 keV Ne^+^, fluence: 1.94 × 10^16^ ions/cm^2^, FoV 16 × 16 μm^2^. Scale bar: 2 μm.

Beneficial to preserving structural details in EM images, TEM and BSE analyses were performed first and destructive SIMS imaging later. As the sample surface was altered after obtaining secondary ion maps, it was necessary to adjust the EM image to the SIMS image. EM images were transformed to an abundant sodium ion signal (Figures 1a and 3a) that reveals a cellular contour. This was done manually in TrakEM2^48^ (Fiji) using nonlinear transformation. Prior to correlation with EM images, SIMS images were resized to 2048 × 2048 pixels. Transformed EM maps and the Fe ion signal were manually aligned in Adobe Illustrator (v. CC 2019, Adobe Inc., 2019. *Adobe Illustrator*, Available at: https://adobe.com/products/illustrator).

## Results and Discussion

### TEM and NanoSIMS Correlative Localization of Iron in Alveolar Macrophages

Macrophages are phagocytic cells of the innate immune system. They have an important role in iron metabolism as they are involved in processes such as production of red blood cells, iron content recycling of the red blood cells, scavenging free heme, and detoxing the free heme. Metabolism of iron is strictly controlled in order to prevent its accumulation leading to cytotoxicity. Moreover, iron is required for heme and Fe–S cluster synthesis as well as metalloproteins to function in essential processes such as oxygen carrying and storage, cellular metabolism, and immune response. Cellular metabolism of iron involves four major steps: uptake, utilization, storage, and export. Following its uptake inside the cell, iron exists as ferrous (Fe^2+^) and ferric (Fe^3+^). The bioavailable and toxic form ferrous iron (Fe^2+^) is used for heme and Fe–S cluster synthesis, and energy production via cytochrome C in mitochondria also requires iron.^49^ In cytoplasm, Fe^2+^ and Fe^3+^ form redox-active iron complexes that are collectively named labile iron pool (LIP).^50^ From the LIP, iron can be used for current needs of cells or stored for later utilization or in a case of a high intracellular iron content exported to prevent cytotoxicity. An intracellular storage of insoluble nontoxic iron (Fe^3+^) is done via a ferritin nanocage. This cytosolic heteropolymer keeps iron always bioavailable by converting ferric iron to its ferrous oxidative state.^49^ In order to localize iron within cells, originating from the LIP or ferritin nanocage, first TEM imaging was done on alveolar macrophages previously exposed to iron and chemically fixed as described in the Experimental Section. Afterward, NanoSIMS analysis was performed on the same ROI with an O^–^ primary ion beam of approximately 250 nm in diameter generating positive secondary ions. The correlation between TEM and SIMS subcellular analysis is shown in Figure 1 (two additional examples are represented in Figures S2 and S3). An overlay of iron signal (Figure 1b) and a corresponding TEM image (Figure 1c) are shown in Figure 1d displaying accumulation of iron in specific subcellular structures. Shown in Figure 1d are two excerpts as examples showing the accumulation of iron inside the mitochondria (on the left) and vacuole-like compartments (on the right). As expected, untreated NR8383 cells as a control group did not give rise to a NanoSIMS iron signal as shown in Figure S6, confirming that the ^56^Fe^+^ signal in treated cells is present as a consequence from their exposure to ammonium iron(III) citrate during sample preparation.

In Figure 2, we summarize selected ROIs from three analyzed cells (outlined in Figure S4) where the presence of iron was detected by NanoSIMS and showed sequestration of iron in mitochondria and vacuole-like organelles. Some of the chosen ROIs correspond to mitochondria (Figure 2a). The NanoSIMS detection of mitochondrial iron is in accordance with the literature, as mitochondria are responsible for the production of heme and more interestingly most of the inorganic cofactor Fe–S clusters that are needed for energy production, metalloprotein function, and immunity. Because of the high demand of iron for mitochondrial processes, the majority of the LIP is trafficked to these organelles.^49^ Additionally, for the sake of not relying purely on the LIP and cytoplasmic iron from the ferritin nanocage, mitochondria can store their own ferritin as well.^51^ Besides cytoplasm and mitochondria, the nucleus can also contain iron from the LIP. Finally, lysosomes, important subcellular compartments responsible for proteolysis of a vast number of proteins such as metalloproteins, can also have high concentrations of labile iron. Moreover, lysosomes can hold some of the ferritin, most likely in iron-loaded cells. Figure 2b indicates sequestration of iron within vacuole-like organelles containing a portion of heterogeneous material. These vacuoles could be possibly lysosomes^52,53^ but also lipid droplets. Yet, from our TEM data it is not completely clear that these subcellular structures are vacuoles, lysosomes, and/or lipid droplets. More detailed TEM analysis as well as staining of the sample with lysosomal markers for subsequent confocal microscopy could provide an answer as has been done previously,^53^ yet this was beyond the scope of the present study. It should be noted that care needs to be always taken when interpreting the results from EM-SIMS correlative studies. For example, the structural details provided by a single TEM image represent the projected information of the 3D sample volume into a 2D image. In order to extract complete 3D structural information, a tilt series of images is needed (tomography approach).^54^ Moreover, a single SIMS 2D chemical image provides compositional information only from the surface near the region of the sample (a few nanometers to tens of nanometers in depth, depending on the selected beam parameters). Therefore, 3D SIMS information can only be extracted by acquiring several 2D chemical maps while removing the sample material layer by layer.^55^

In this study, we employed the duoplasmatron oxygen ion source that has a limited lateral resolution (few hundreds of nanometers). The newer NanoSIMS instruments are equipped with an improved oxygen source, the radiofrequency (RF) plasma oxygen source that can be focused down to 40 nm. It has been shown that it is possible to analyze metals such as iron in biological systems at high-sensitivity and high-spatial resolution (down to 37 nm).^56−58^ Utilization of the new oxygen primary ion source would be a next step to take in further NanoSIMS studies focusing on localization of iron at the nanometer scale.

### BSE and HIM-SIMS Imaging of Iron in Alveolar Macrophages

The HIM-SIMS instrument can provide structural information based on a secondary electron (SE) signal by usually employing the He^+^ primary ion beam for sample irradiation. HIM has several advantages in comparison with SEM such as better surface detail and larger depth of field.^59^ Sato *et al.* analyzed unstained and uncoated kidney tissue sections with HIM and showed the possibility to visualize red blood cells and some subcellular structures such as the nucleus and nucleoli. Yet, identification of smaller subcellular compartments such as mitochondria and lysosomes was uncertain.^60^ In our study, we found imaging of flat, nonconductive samples such as resin-embedded cells quite difficult due to charge buildup. For this reason, it was necessary to sputter-coat samples with a conductive material such as gold. Typical for HIM imaging is that the SEs are emitted close to the spot of the beam impact and the SE yield is high, both allowing to attain a high surface detail.^36^ Thus, HIM provided structural information based on topographic features of the section. Yet, discerning different types of organelles other than the nucleus was not attainable (Figures S7 and S9). Therefore, prior to SIMS analysis, structural information was obtained with BSE imaging ex situ where the generation of contrast depends on the atomic number of atoms in a specimen. Osmium and uranium were used as stains and as they have a high atomic number with more electrons around the nucleus, and their presence allowed more incident electrons to be backscattered; thus, a good contrast was attained in BSE images. Yet, in our study, the quality of BSE imaging could be improved if sections were left uncoated as these samples did not show a significant charge built up during BSE image acquisition (Figure S8). Thus, for future experiments, we suggest BSE imaging prior to coating. Figure 3 shows the summarized BSE-HIM-SIMS correlative analysis of iron in the NR8383 cell. As in the TEM-NanoSIMS experiment, we needed to correlate structural and chemical data and first transform the BSE image to fit the SIMS abundant sodium signal that was used as a cell “fingerprint”. Afterward, the transformed BSE image was correlated with the binned iron signal.

We found that the localization of Fe in vacuoles–lysosomes was in agreement with the TEM-NanoSIMS results. HIM-SIMS provides significantly higher spatial resolution than the duoplasmatron oxygen source present on the NanoSIMS instrument. Yet, due to the inertness of the Ne primary ions used for analysis, the^56^Fe^+^ secondary ion yield was lower in comparison with the one obtained with the reactive oxygen source in the NanoSIMS. Integration of neighboring pixels (2 × 2 binning) helped to improve the contrast for HIM-SIMS iron detection. The post-acquisition image processing by binning degraded lateral resolution by a factor of 2; still, the spatial resolution was better than what the duoplasmatron oxygen source can provide. Control cells were also analyzed in a comparative manner, and they did not show accumulation of iron as shown in Figure S9. Due to instrumental constraints in HIM-SIMS, the double-focusing magnetic sector mass analyzer provides a moderate mass resolving power (at FWHM, it is around 500 at an overall transmission >40%), thus not allowing to mass resolve isobaric interferences ^56^Fe and ^40^Ca^16^O, which requires a mass resolving power *m*/Δ*m* ≈ 2500.^55^ As we were unable to resolve this interference, we acquired ion maps of ^56^Fe^+^ and ^40^Ca^+^ to investigate if their distribution across the cells would be different (Figure S10). This provided insights that we were not imaging signals from ^56^Fe^+^ and ^40^Ca^16^O^+^ ion species simultaneously. As it is shown in Figure S10, we used the endogenous CN^–^ ion signal for the structural information of the cells (Figure S10a). Figure S10b and S10c shows maps for ^40^Ca^+^ and ^56^Fe^+^ ion species, respectively. In Figure S10d and S10e where their signals are binned, it is evident that these two secondary ion species do not colocalize; therefore, we can exclude the contribution of ^40^Ca^16^O^+^ to the targeted ^56^Fe^+^ signal. As mentioned above, HIM-SIMS employs a Ne^+^ primary ion beam to generate both positive and negative secondary ions, by simply switching the sample bias from +500 to −500 V and hence inverting the electric extraction field between the sample and the extraction optics. The opportunity to perform imaging in both polarities without switching between the ion sources, as it is required on the NanoSIMS instrument, is a big benefit of the HIM-SIMS instrument. One of the resulting advantages is the possibility to obtain morphological information based on the endogenous ^12^C^14^N^–^ chemical signal that originates from all carbon- and nitrogen-containing molecules in the specimen when analyzing in the negative polarity. By easily switching the polarity to positive mode, one can obtain the spatial distribution of positive secondary ions during the same experiment. This is incredibly valuable in situations where no structural information has been obtained prior to chemical imaging. When the ^12^C^14^N^–^ signal is used for structural characterization, one has to be aware that it is possible to visualize, e.g., nucleus, nucleoli, and mitochondria (subcellular structures with high contents of C and N), but compartments that appear electrolucent in EM such as lysosomes, vacuoles, phagosomes, and lipid droplets cannot be distinguished (Figure S11). In our study, we performed SIMS imaging in positive polarity first to analyze the less abundant Fe species. Following the initial analysis, the polarity was changed to negative in order to acquire a ^12^C^14^N^–^ ion map. It is noteworthy to mention that the FoV for the ^12^C^14^N^–^ image was slightly reduced (1–2 μm) in order to avoid imaging sample damage (crater edges) generated during previous analysis in a positive polarity. In the CN-Fe overlay, it is shown that subcellular compartments lacking a CN signal (looking empty in the ^12^C^14^N^–^ ion map) contain iron. This is compatible with the BSE-Fe overlay shown in Figure 3f, where electron-lucent organelles, such as vacuoles, contain iron.

In the future, there are several aspects that should be addressed to improve correlative subcellular EM-SIMS analysis of iron. Sample preparation of biological specimens is rather challenging as both EM and SIMS instruments require an ultra-high-vacuum environment. Thus, for example, cells that are rich in water had to be preserved in order to resemble as close as possible their native state. In order to achieve this, we had to chemically fix, dehydrate, resin-embed, and section the sample for correlative EM-SIMS imaging.^61^ During these processes, water-soluble analytes such as Fe^2+^/Fe^3+^ ions can be redistributed or even washed out from the cell, altering the chemical information from its native state. In this context, cryofixation would be a more appropriate approach to fix the specimen and the sample to be analyzed in a frozen hydrated state.^62^ A new tool, npSCOPE instrument, has been developed that allows analyses under cryo conditions. Additionally, it integrates the possibility for scanning transmission ion microscopy (STIM) using a 2D position sensitive detector in HIM-SIMS. Here, the He^+^ beam can pass through thin samples such as sections prepared for TEM. Primary ions will interact with the specimen moiety, and a substantial fraction of them will pass through the sample providing structural information.^63^ Therefore, acquisition of structural and chemical data in situ for thin flat cryopreserved biosamples is possible.^64^ Regarding sensitivity of the SIMS analysis mode, it is feasible to implement flooding with reactive oxygen species^65,66^ during analysis that can enhance the useful yield of positive secondary ions such as iron ion species.

## Conclusions

In this study, we analyzed iron in alveolar macrophages with two SIMS instruments, NanoSIMS and HIM-SIMS, both equipped with a magnetic sector mass analyzer. As these techniques can provide structural information only based on the endogenous ^12^C^14^N^–^ secondary ion signal, more detailed information regarding cell structure was needed to be acquired prior to the destructive SIMS analyses. For this reason, we correlated TEM data with chemical information obtained by NanoSIMS and BSE images with HIM-SIMS results. The NanoSIMS instrument allowed high-mass-resolution imaging and—due to the reactive nature of the oxygen primary ions, which can easily ionize electropositive elements like metals—high-sensitivity analysis. Yet, the lateral resolution was limited to approximately 250 nm as a consequence of utilizing the duoplasmatron oxygen source. With HIM-SIMS, analysis at a sub-20 nm lateral resolution can be achieved; however, the inertness of the Ne^+^ primary ion species restricts the sensitivity of iron detection. Moreover, due to the moderate mass resolving power of HIM-SIMS, only elemental analysis was possible. The TEM-NanoSIMS experiments revealed accumulation of iron mostly in mitochondria and vacuole-like organelles, while BSE-HIM-SIMS correlative analysis showed the iron content within vacuole-like organelles. Our study demonstrates the future potential and applicability of high-resolution imaging techniques in mapping of iron at the subcellular level within various biological samples of interest. We also propose methodological suggestions that can improve analysis of iron in the future.

## Supporting Information Available

Additional experimental details on imaging methodologies and imaging data of correlative TEM-NanoSIMS and SEM-HIM-SIMS analyses (PDF)
