# Supplementary material for: Correlative High-Resolution Imaging of Iron Uptake in Lung Macrophages
Source: Anal Chem. 2022 Sep 7;94(37):12798–806. doi: 10.1021/acs.analchem.2c02675 (PMC9494303; doi:10.1021/acs.analchem.2c02675)
Supplement: Supplementary file 1 — ac2c02675_si_001.pdf [file ac2c02675_si_001.pdf]

Supporting Information

Correlative high-resolution imaging of iron uptake in lung macrophages

Jelena Lovrić[1], Neda Najafinobar[2†], Michael E. Kurczy[1], Olivier De Castro[3], Antje Biesemeier[3], Lena von Sydow[2], Magnus Klarqvist[4], Tom Wirtz[3] & Per Malmberg[5*]

[1] DMPK, Research and Early Development, Cardiovascular, Renal and Metabolism, BioPharmaceuticals R&D, AstraZeneca, SE-431 50 Gothenburg, Sweden

[2] Medicinal Chemistry, Research and Early Development, Respiratory and Immunology, BioPharmaceuticals R&D, AstraZeneca, SE-431 50 Gothenburg, Sweden

[3] Advanced Instrumentation for Nano-Analytics (AINA), MRT Department, Luxembourg Institute of Science and Technology (LIST), L-4422 Belvaux, Luxembourg

[4] Early Product Development, Pharm Sci, IMED Biotech Unit, AstraZeneca, SE-431 50 Gothenburg, Sweden

[5] Department of Chemistry and Chemical Engineering, Chalmers University of Technology, SE-412 96 Gothenburg, Sweden

[*] Corresponding author:

Department of Chemistry and Chemical Engineering, Chalmers University of Technology, SE-412 96 Gothenburg, Sweden; http://orcid.org/0000-0002-6487-7851; Phone: +46317728321; Email: malmper@chalmers.se

[†] Present address:

Clinical Pharmacology and Safety Sciences, Business Planning and Operations, Biopharmaceuticals R&D, AstraZeneca, SE-431 50 Gothenburg, Sweden

Table of Contents:

Figure S-1. Methodology of sectioning for TEM imaging followed by NanoSIMS analysis……**S3**

Figure S-2. Correlative TEM and NanoSIMS imaging of iron in chemically fixed alveolar macrophages previously exposed to 500 μM ammonium iron (III) citrate……**S4**

Figure S-3. Correlative TEM and NanoSIMS imaging of iron in chemically fixed alveolar macrophages previously exposed to 500 μM ammonium iron (III) citrate……**S5**

Figure S-4. NanoSIMS images of iron in chemically fixed alveolar macrophages previously exposed to 500 μM ammonium iron (III) citrate. ……………………………………………………**S6**

Figure S-5. Selected ROIs from NanoSIMS images of iron in chemically fixed alveolar macrophages previously exposed to 500 μM ammonium iron (III) citrate ……**S7**

Figure S-6. NanoSIMS analysis of negative control alveolar macrophages………………………**S8**

Figure S-7. HIM-SIMS data of sectioned and gold coated NR8383 cells previously treated with 500 μM ammonium iron (III) citrate ……**S9**

Figure S-8. Comparison between BSE imaging of alveolar macrophages in section with and without gold coating ……**S10**

Figure S-9. HIM-SIMS analysis of negative control alveolar macrophages ……**S11**

Figure S-10. HIM-SIMS analysis of iron treated alveolar macrophages. Comparison between distribution of $^{40}Ca^{+}$ and $^{56}Fe^{+}$ signals ……...**S12**

Figure S-11. Iron treated alveolar macrophages, methodology for correlation between endogenous $^{12}C^{14}N^{-}$ signal revealing structural information and binned $^{56}Fe^{+}$ ion signal……**S13**

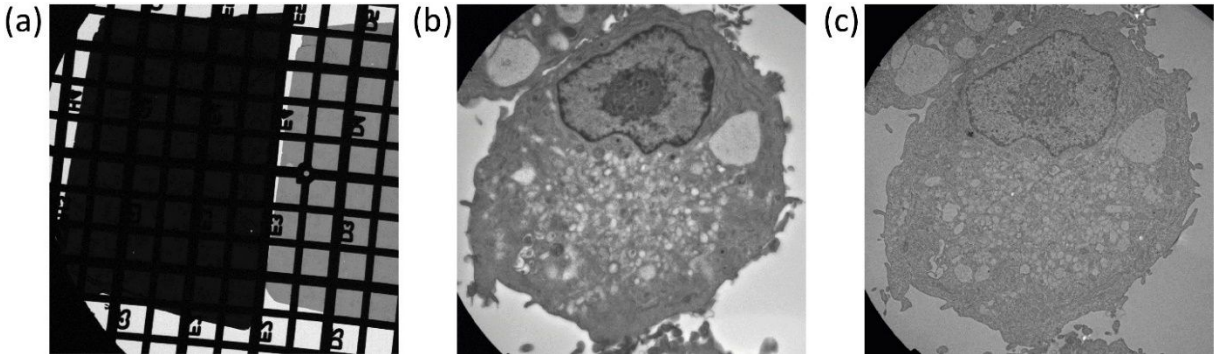

Figure S-1. Methodology of sectioning for TEM imaging followed by NanoSIMS analysis. (a) Low magnification TEM image showing two sections on formvar coated copper grid, on left (darker) of 300 *n*m thickness for NanoSIMS analysis and on right (lighter) of 70 *n*m thickness for TEM imaging; (b) TEM image of alveolar macrophage from 300 *n*m section showing lower quality of a cellular detail; (c) TEM image of alveolar macrophage from 70 *n*m section showing higher quality of cellular detail used for structural information.

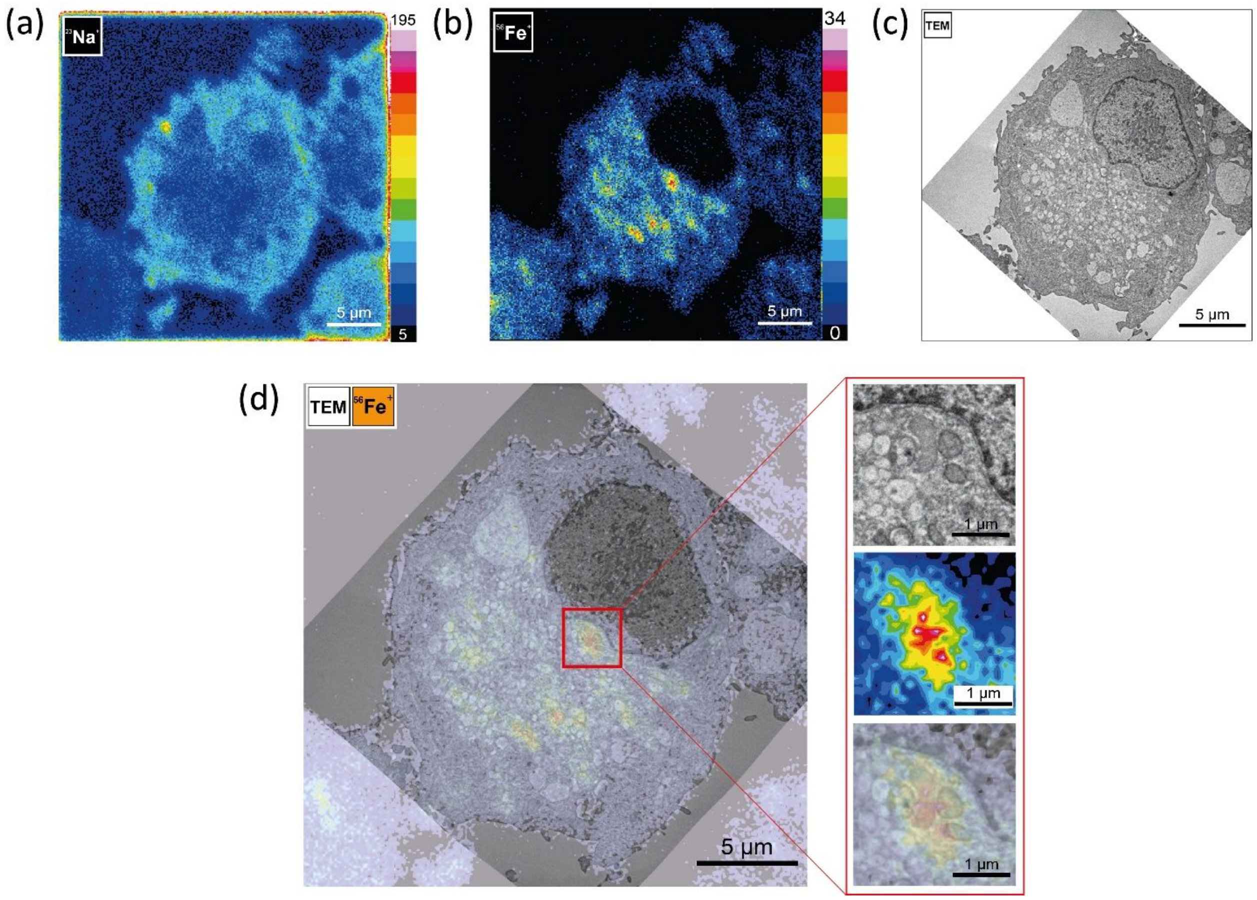

Figure S-2. Correlative TEM and NanoSIMS imaging of iron in chemically fixed alveolar macrophages previously exposed to 500 μM ammonium iron (III) citrate. NanoSIMS imaging: (a) Ion map of $^{23}Na^{+}$ revealing cellular contour; (b) $^{56}Fe^{+}$ ion map, for both NanoSIMS images: 16 *k*eV O^{-}, Fluence: 11.47 × 10^{16} ions/cm^2, FoV: 30 × 30 μm^2, number of planes 40; (c) Corresponding TEM image; (d) An overlay of TEM and $^{56}Fe^{+}$ signal to correlate structural and chemical information. ROI from TEM image, $^{56}Fe^{+}$ ion map and from their overlay, showing localization of iron in mitochondria. Scale bars: 1 μm and 5 μm.

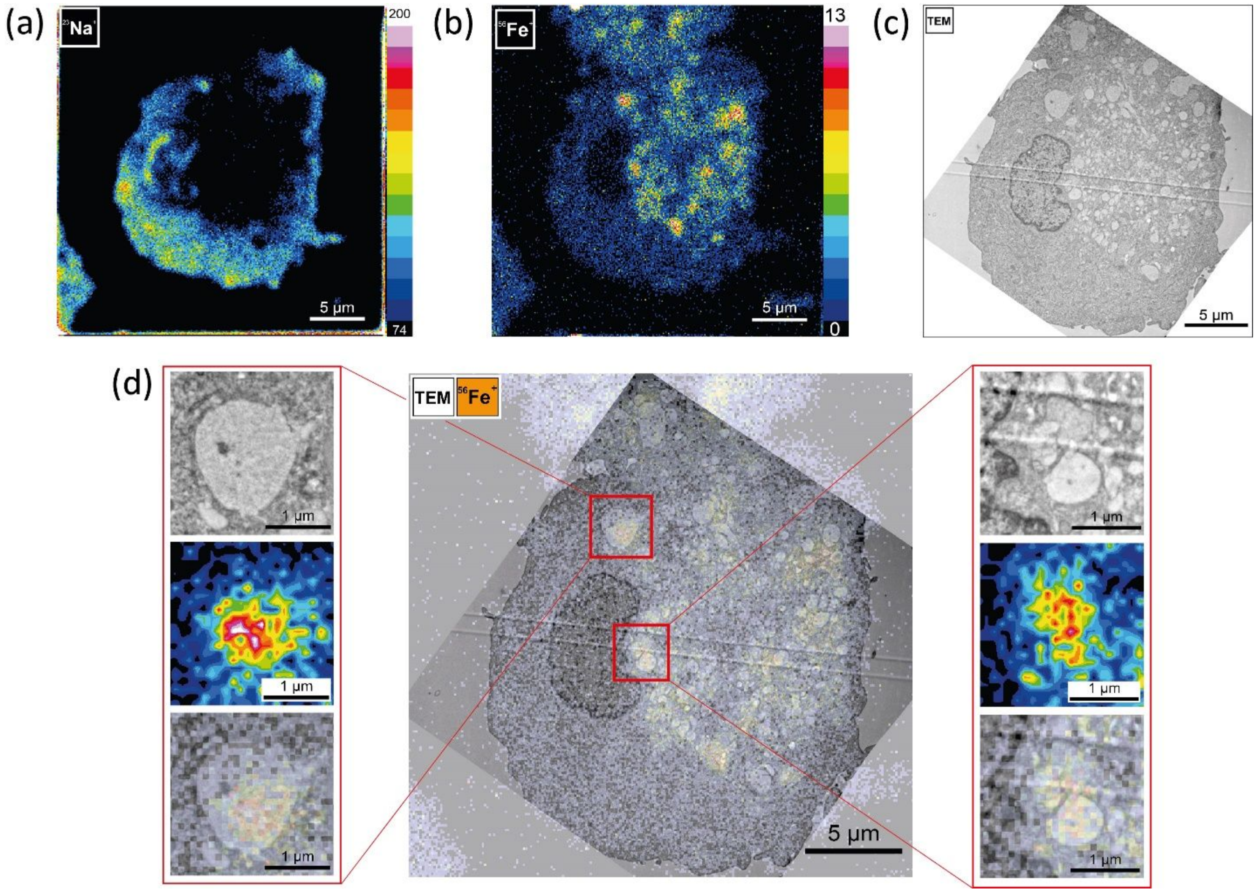

Figure S-3. Correlative TEM and NanoSIMS imaging of iron in chemically fixed alveolar macrophages previously exposed to 500 μM ammonium iron (III) citrate. NanoSIMS imaging: (a) Ion map of $^{23}Na^{+}$ revealing cellular contour; (b) $^{56}Fe^{+}$ ion map, for both NanoSIMS images: 16 *k*eV O^{-}, Fluence: 4.7×10^{16} ions/cm^{2}, FoV: 30×30 μm^{2}, number of planes 12; (c) Corresponding TEM image; (d) An overlay of TEM and $^{56}Fe^{+}$ signal to correlate structural and chemical information. Two ROIs from TEM image, $^{56}Fe^{+}$ ion map and from their overlay, showing localization of iron in vacuole like organelles. Scale bars: 1 μm and 5 μm.

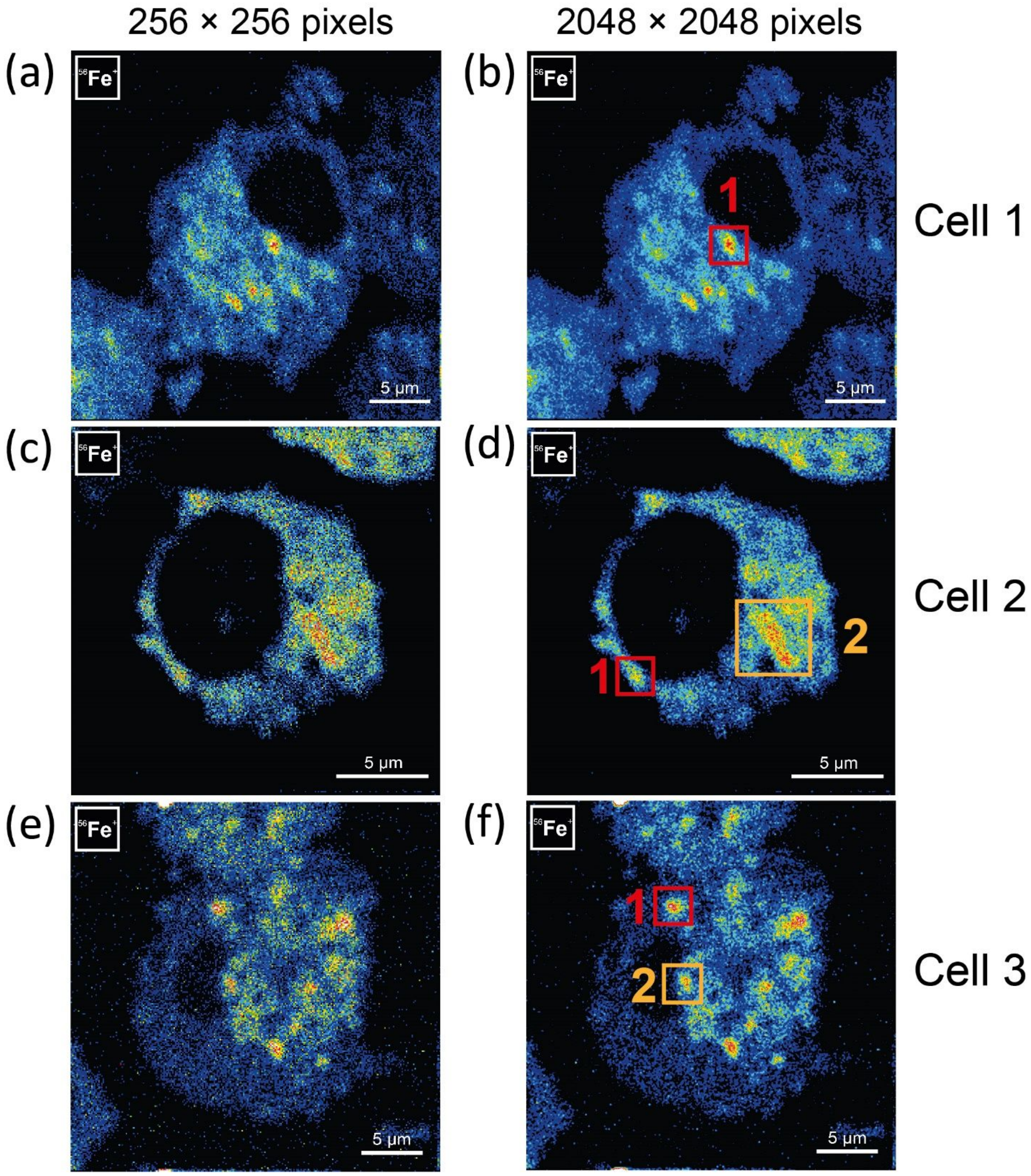

Figure S-4. NanoSIMS images of iron in chemically fixed alveolar macrophages previously exposed to 500 μM ammonium iron (III) citrate. Cell 1: (a), (b) 16 *k*eV O^-, Fluence: 11.47 × 10^{16} ions/cm^2, FoV: 30 × 30 μm^2, number of planes 40, (a) 256 × 256 pixels, (b) 2048 × 2048 pixels; Cell 2: (c), (d) 16 *k*eV O^-, Fluence: 36.11 × 10^{16} ions/cm^2, FoV: 20 × 20 μm^2, number of planes: 41, (c) 256 × 256 pixels, (d) 2048 × 2048 pixels; Cell 3: (e), (f) 16 *k*eV O^-, Fluence: 4.7 × 10^{16} ions/cm^2, FoV: 30 × 30 μm^2, number of planes 12; (e) 256 × 256 pixels, (f) 2048 × 2048 pixels. Selected ROIs were obtained from ion images with 2048 × 2048 pixels in size (Figures S-4b, S4-d and S-4f) and they are labelled with number 1 (red square) and number 2 (orange square). Scale bar: 5 μm.

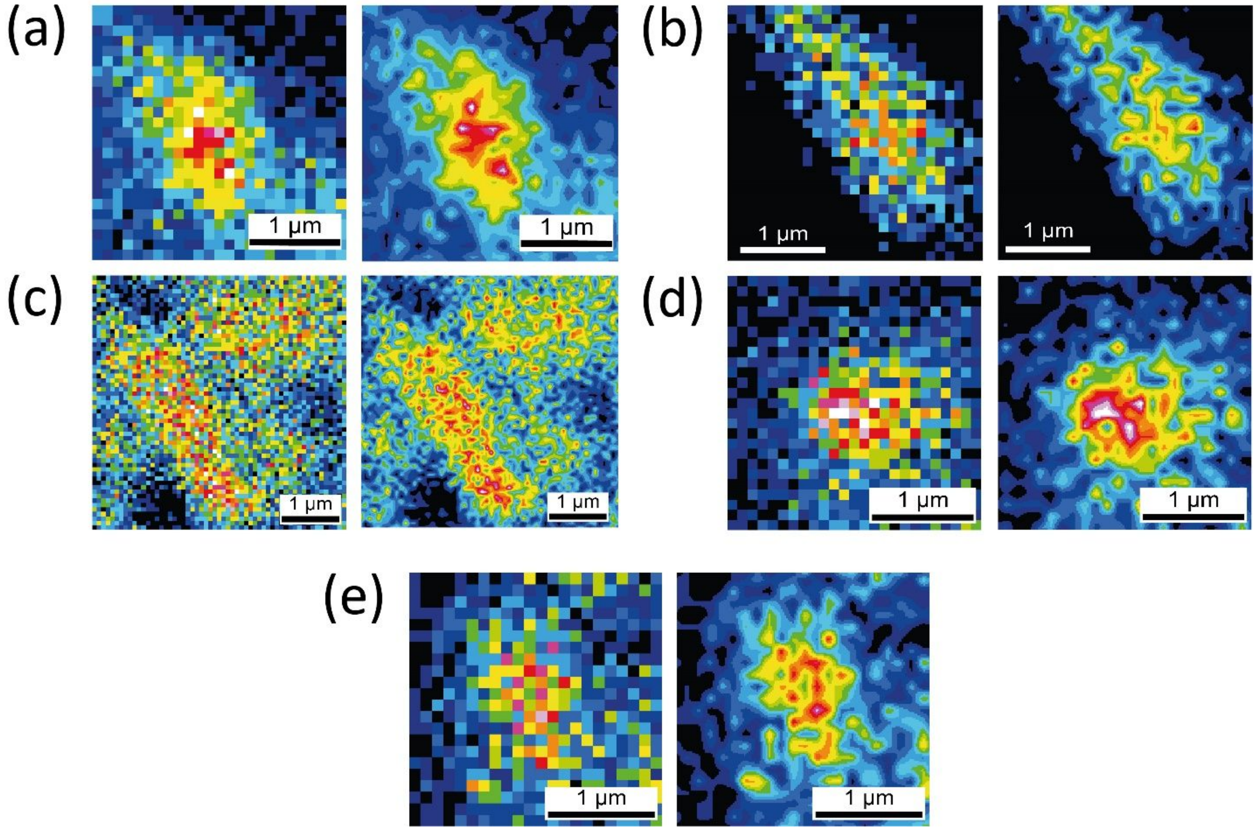

Figure S-5. Selected ROIs from NanoSIMS images of iron in chemically fixed alveolar macrophages previously exposed to 500 μM ammonium iron (III) citrate. (a) ROI 1 from Cell 1 (Figures S-4a and S-4b), FoV: 2.8 × 2.8 μm^2, left image: 24 × 24 pixels, right image: 191 × 191 pixels; (b) ROI 1 from Cell 2 (Figures S-4c and S-4d), FoV: 3 × 3 μm^2, left image: 38 × 38 pixels, right image: 307 × 307 pixels; (c) ROI 2 from Cell 2 (Figures S-4c and S-4d), FoV: 4.5 × 4.5 μm^2, left image: 58 × 58 pixels, right image: 460 × 460 pixels; (d) ROI 1 from Cell 3 (Figures S-4e and S-4f), FoV: 2.5 × 2.5 μm^2, left image: 21 × 21 pixels, right image: 171 × 171 pixels; (e) ROI 2 from Cell 3 (Figures S-4e and S-4f), FoV: 2.4 × 2.4 μm^2, left image: 21 × 21 pixels, right image: 164 × 164 pixels. Scale bar: 1 μm.

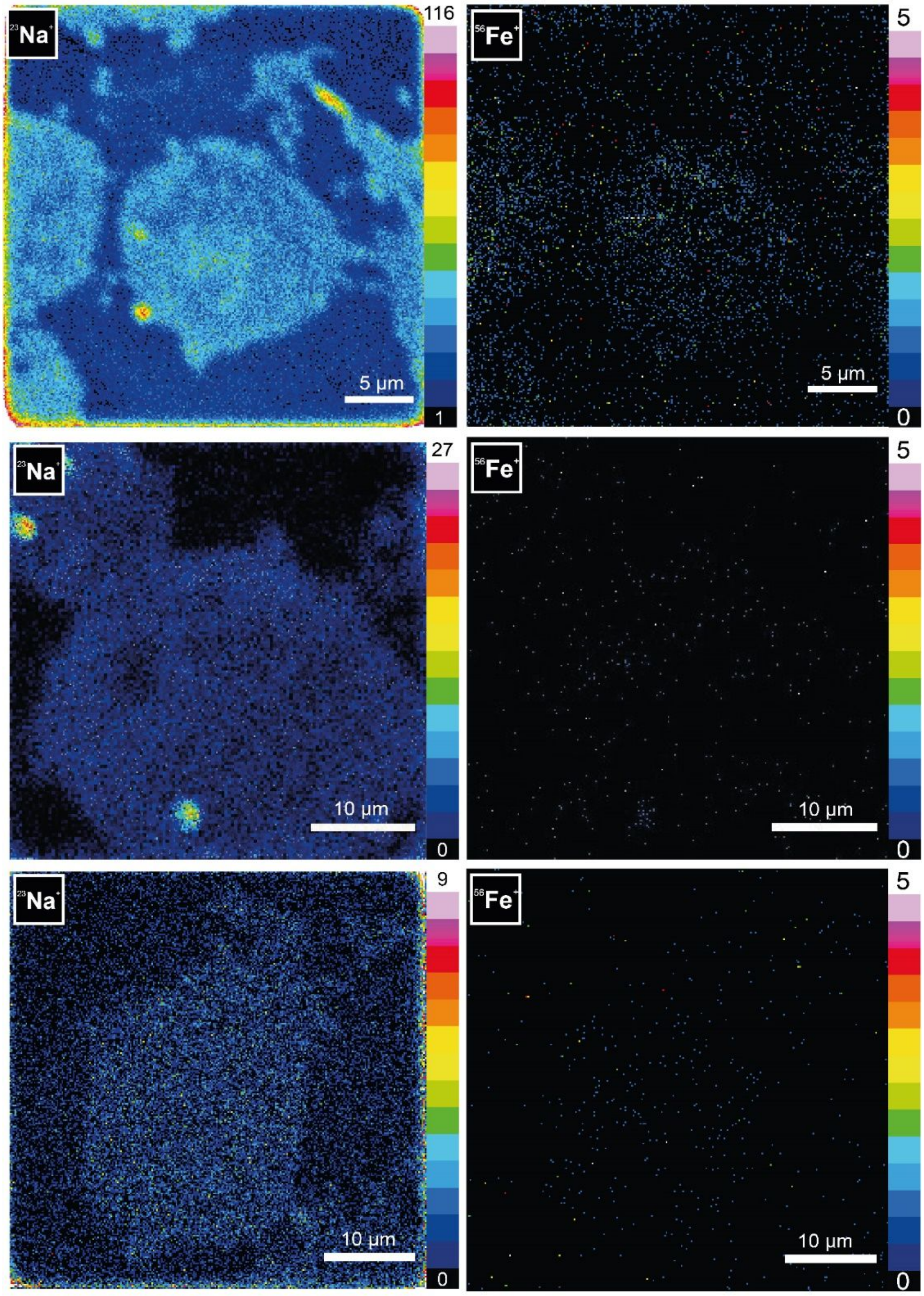

Figure S-6. NanoSIMS analysis of negative control alveolar macrophages. On left, a map of $^{23}Na^{+}$ revealing cellular contour; on right, $^{56}Fe^{+}$ ion map. NanoSIMS images: 16 *k*eV O^{-}; Top row: Fluence: 5,87 × 10^{16} ions/cm^2, FoV: 30 × 30 μm^2, number of planes: 15; Middle row: Fluence: 1,1 × 10^{16} ions/cm^2, FoV: 40 × 40 μm^2, number of planes: 5; Bottom row: Fluence: 1,74 × 10^{16} ions/cm^2, FoV: 45 × 45 μm^2, number of planes: 10. Scale bars: 5 μm and 10 μm.

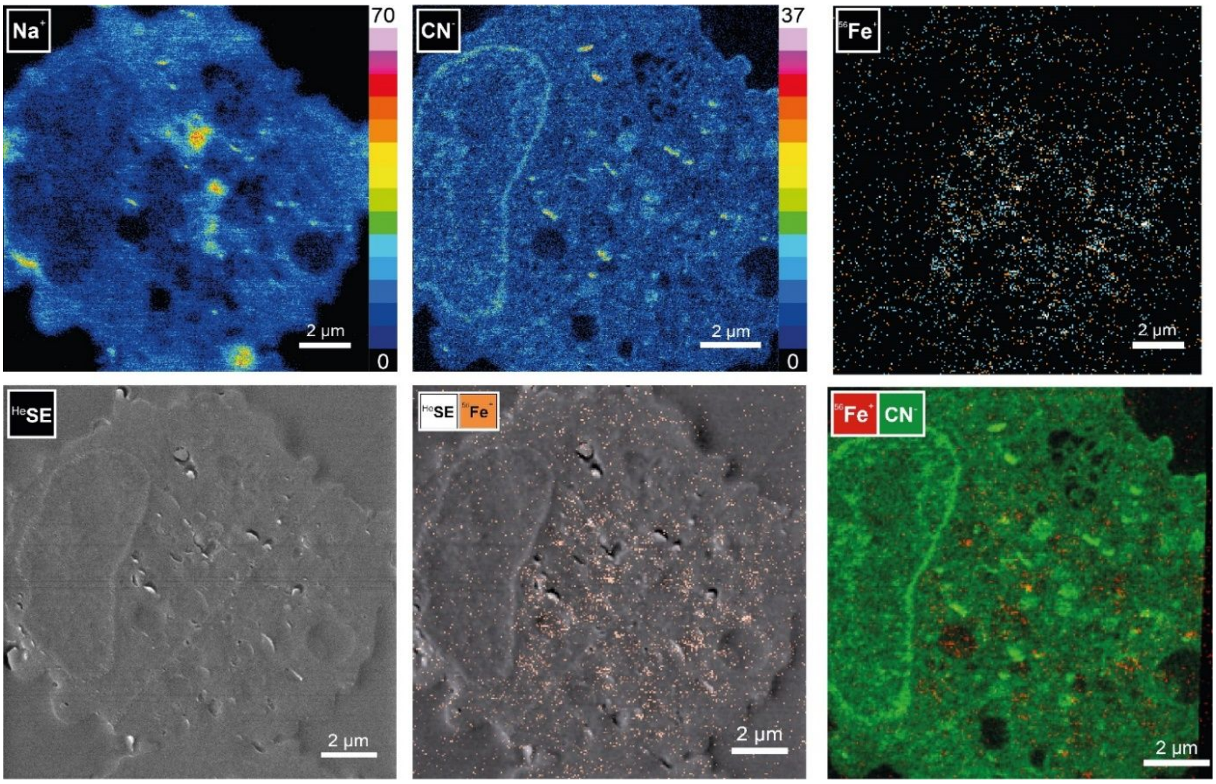

Figure S-7. HIM-SIMS data of sectioned and gold coated NR8383 cells previously treated with 500 μM ammonium iron (III) citrate. HIM does not provide necessary structural information of stained cells in a section. Therefore, it is needed to perform SEM imaging where heavy metal stains can be visualized in cells. Top row: $^{23}Na^{+}$ image, $^{12}C^{14}N^{-}$ ion map, binned $^{56}Fe^{+}$ ion signal (X,Y shrink factor: 2; bin method: sum); Bottom row: HIM image, overlay of HIM image and binned $^{56}Fe^{+}$ ion signal, overlay of $^{12}C^{14}N^{-}$ and binned $^{56}Fe^{+}$ ion signal. Imaging parameters for HIM image: 20 *k*eV He^{+}, 1024 × 1024 pixels, 10 μs scan dwell time, FoV 14 × 14 μm^2. Imaging parameters for HIM-SIMS analysis: 20 *k*eV Ne^{+}, Negative polarity: fluence: 1.7 × 10^{16} ions/cm^2, FoV 12 × 12 μm^2; Positive polarity: fluence: 2.2 × 10^{16} ions/cm^2, FoV 14 × 14 μm^2. Scale bar: 2 μm.

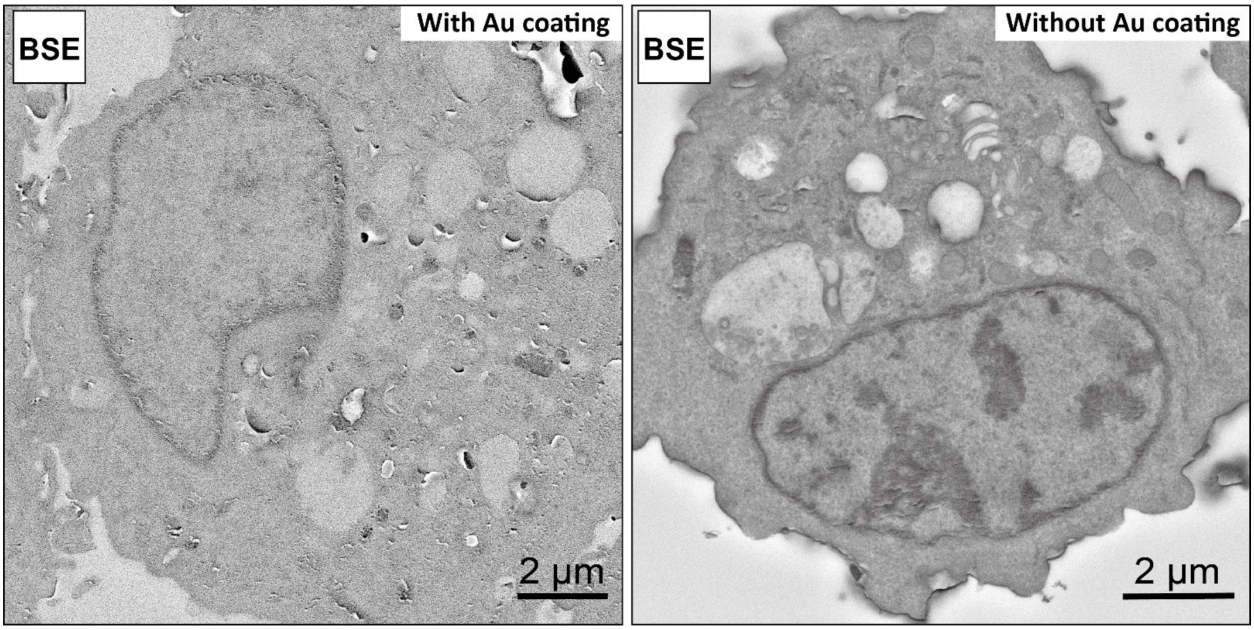

Figure S-8. Comparison between BSE imaging of alveolar macrophages in section that was gold coated prior to BSE analysis (left) and without coating (right). Scale bar: 2 μm.

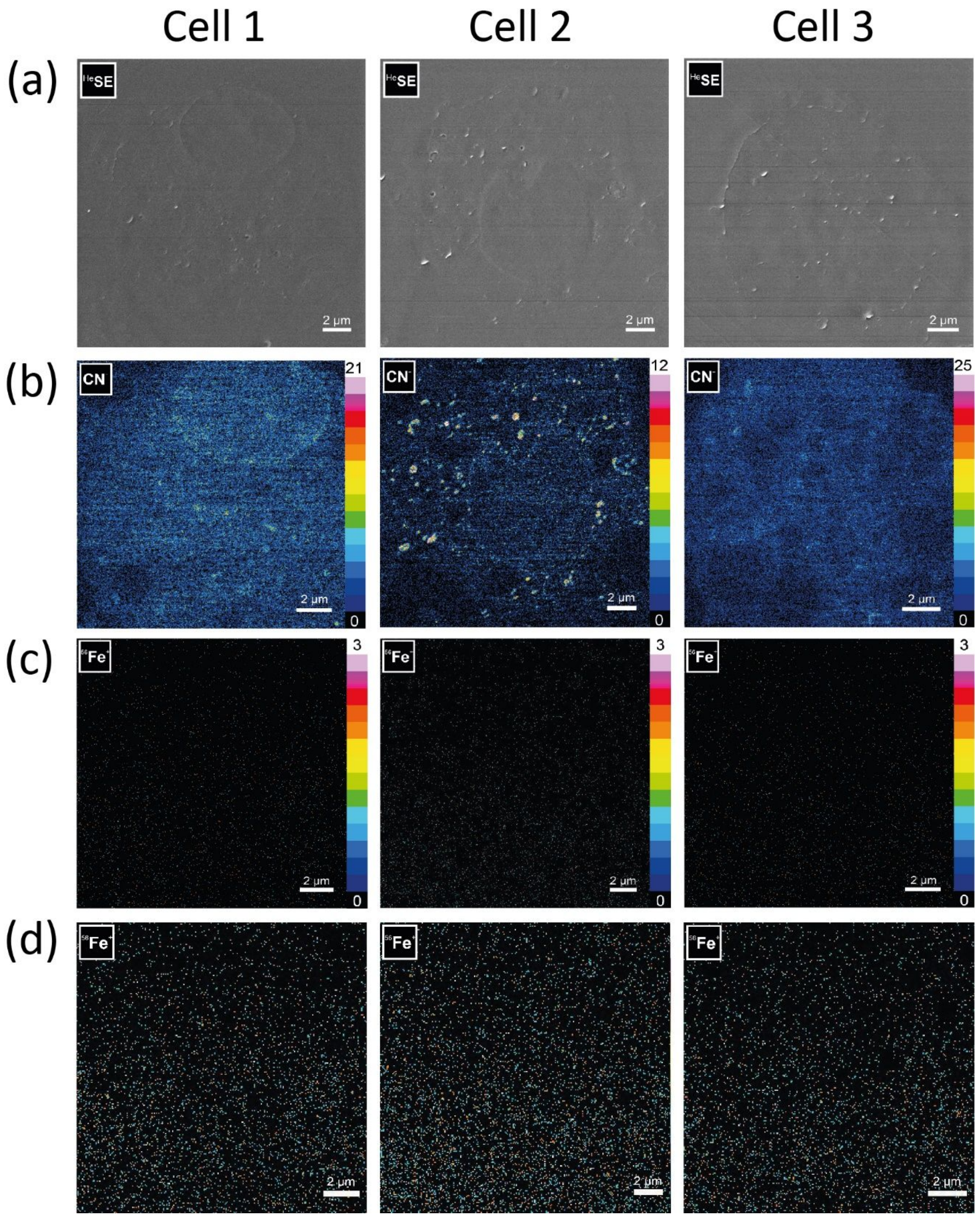

Figure S-9. HIM-SIMS analysis of negative control alveolar macrophages. (a) HIM image, 20 *k*eV He^+, 1024 × 1024 pixels, 10 μs scan dwell time; (b) $^{12}C^{14}N^-$ signal revealing cellular contour; (c) $^{56}Fe^+$ ion image; (d) $^{56}Fe^+$ ion signal binned (X,Y shrink factor: 2; bin method: sum). HIM-SIMS imaging parameters: 20 *k*eV Ne^+; Cell 1 - Negative polarity: Fluence: 1.18 × 10^{16} ions/cm^2, FoV: 15 × 15 μm^2; Positive polarity: Fluence: 1.33 × 10^{16} ions/cm^2, FoV: 16 × 16 μm^2; Cell 2 – Negative polarity: Fluence: 0.85 × 10^{16} ions/cm^2, FoV: 18 × 18 μm^2; Positive polarity: Fluence: 0.74 × 10^{16} ions/cm^2, FoV: 20 × 20 μm^2; Cell 3 – Negative polarity: Fluence: 1.36 × 10^{16} ions/cm^2, FoV: 14 × 14 μm^2; Positive polarity: Fluence: 1.44 × 10^{16} ions/cm^2, FoV: 15 × 15 μm^2. Scale bar: 2 μm.

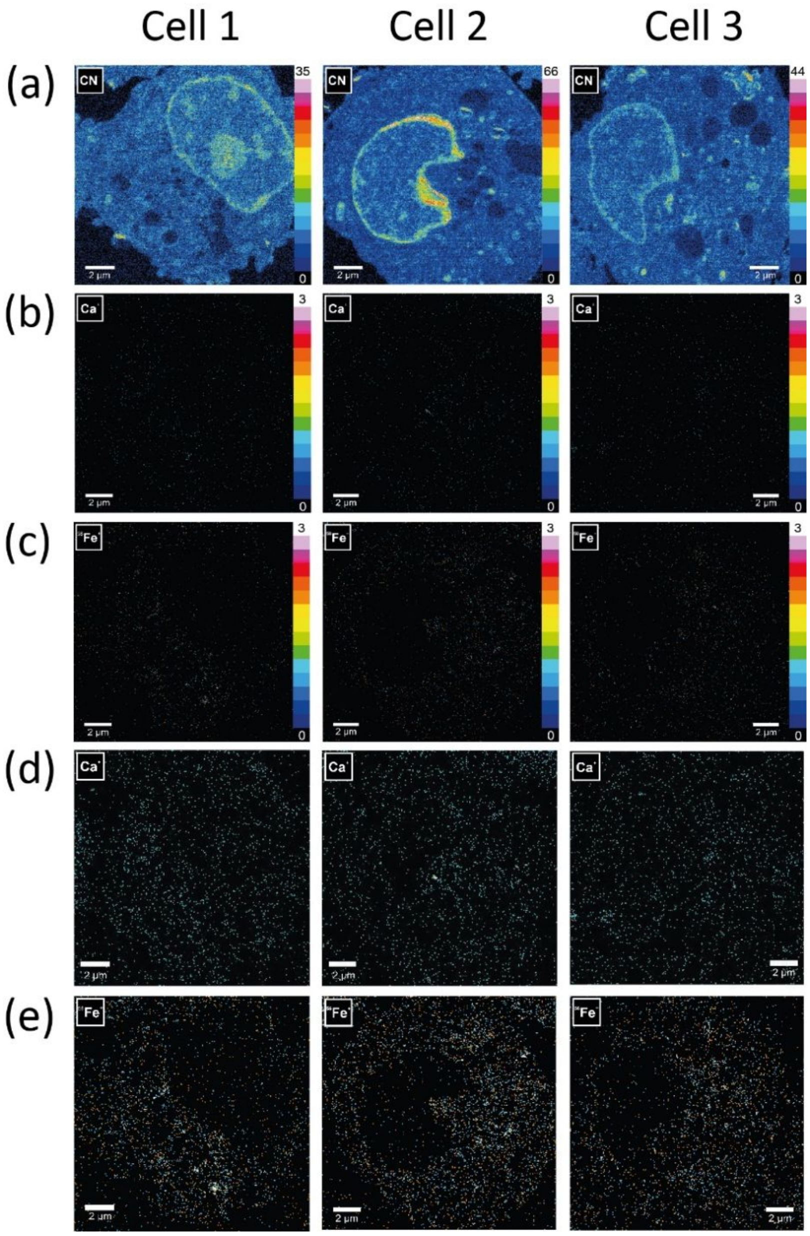

Figure S-10. HIM-SIMS analysis of iron treated alveolar macrophages. Comparison between distribution of $^{40}Ca^{+}$ and $^{56}Fe^{+}$ signals to exclude contribution of $^{40}Ca^{16}O^{+}$ (m/z 56) signal to $^{56}Fe^{+}$ signal. (a) $^{12}C^{14}N^{-}$ signal revealing cellular contour; (b) $^{40}Ca^{+}$ ion map; (c) $^{56}Fe^{+}$ ion image; (d) $^{40}Ca^{+}$ ion signal binned (X,Y shrink factor: 2; bin method: sum); (e) $^{56}Fe^{+}$ ion signal binned (X,Y shrink factor: 2; bin method: sum). Imaging parameters: 20 *k*eV Ne^{+}; Cell 1 - Negative polarity: Fluence: 1.44×10^{16} ions/cm^2, FoV: 15×15 μm^2; Positive polarity: Fluence: 1.94×10^{16} ions/cm^2, FoV: 16×16 μm^2; Cell 2 – Negative polarity: Fluence: 1.86×10^{16} ions/cm^2, FoV: 15×15 μm^2; Positive polarity: Fluence: 1.60×10^{16} ions/cm^2, FoV: 17×17 μm^2; Cell 3 – Negative polarity: Fluence: 1.55×10^{16} ions/cm^2, FoV: 15×15 μm^2; Positive polarity: Fluence: 1.74×10^{16} ions/cm^2, FoV: 17×17 μm^2. Scale bar: 2 μm.

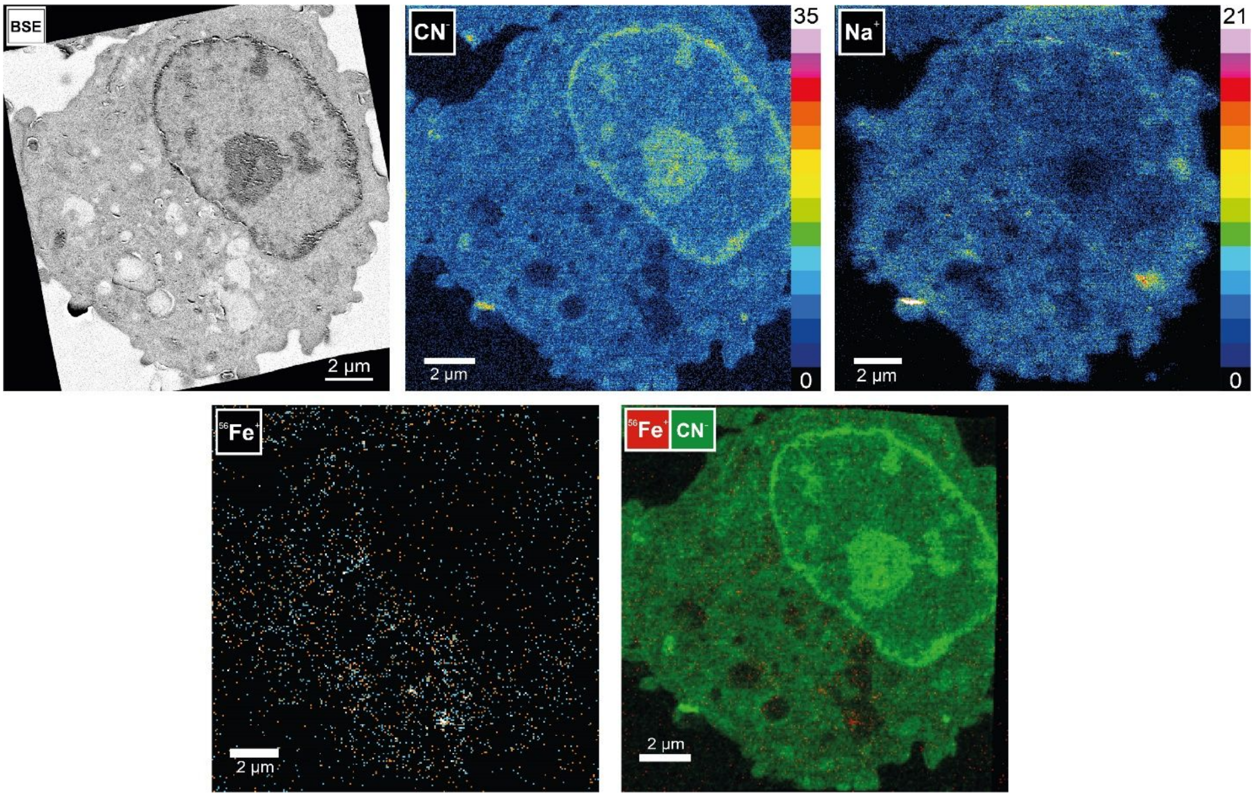

Figure S-11. Iron treated alveolar macrophages, methodology for correlation between endogenous $^{12}C^{14}N^-$ signal revealing structural information and binned $^{56}Fe^+$ ion signal. Top row: BSE image; $^{12}C^{14}N^-$ ion image; $^{23}Na^+$ ion map; Bottom row: binned $^{56}Fe^+$ ion signal; overlay of $^{12}C^{14}N^-$ and binned $^{56}Fe^+$ ion signal (X,Y shrink factor: 2; bin method: sum). HIM-SIMS imaging: 20 *k*eV Ne^+; Negative polarity: Fluence: 1.44×10^{16} ions/cm^2, FoV: 15×15 μm^2; Positive polarity: Fluence: 1.94×10^{16} ions/cm^2, FoV: 16×16 μm^2. Scale bar: 2 μm.
